# Predictors of Anxiety-Induced Sleep Disturbance among in-School Adolescents in Ghana: Evidence from the 2012 Global School-Based Health Survey

**DOI:** 10.3390/bs11020020

**Published:** 2021-02-01

**Authors:** Bright Opoku Ahinkorah, Richard Gyan Aboagye, Francis Arthur-Holmes, Abdul-Aziz Seidu, James Boadu Frimpong, Eugene Budu, Bernard Mensah Amoako, John Elvis Hagan

**Affiliations:** 1 School of Public Health, Faculty of Health, University of Technology Sydney, Sydney, NSW 2007, Australia; brightahinkorah@gmail.com; 2 School of Public Health, University of Health and Allied Sciences, Ho PMB 31, Ghana; raboagye18@sph.uhas.edu.gh; 3 Department of Sociology and Social Policy, Lingnan University, 8 Castle Peak Road, Tuen Mun, Hong Kong; frarthur88@gmail.com; 4 Department of Population and Health, University of Cape Coast, Cape Coast PMB TF0494, Ghana; abdul-aziz.seidu@stu.ucc.edu.gh (A.-A.S.); budueugene@gmail.com (E.B.); 5 College of Public Health, Medical and Veterinary Services, James Cook University, Townsville, QLD 4811, Australia; 6 Department of Health, Physical Education, and Recreation, University of Cape Coast, Cape Coast PMB TF0494, Ghana; frimpongboadujames@gmail.com; 7 Department of Counselling Psychology, University of Education, Winneba, Winneba PMB 25, Ghana; bmamoako@uew.edu.gh; 8 Neurocognition and Action-Biomechanics-Research Group, Faculty of Psychology and Sport Sciences, Bielefeld University, Postfach 10 01 31, 33501 Bielefeld, Germany

**Keywords:** Ghana, in-school adolescents, hunger, loneliness, cognitive-behavioral, suicidal ideations, therapy

## Abstract

(1) *Background:* Psychological problems of adolescents have become a global health and safety concern. Empirical evidence has shown that adolescents experience diverse mental health conditions (e.g., anxiety, depression, and emotional disorders). However, research on anxiety-induced sleep disturbance among in-school adolescents has received less attention, particularly in low- and middle-income countries. This study’s central focus was to examine factors associated with t anxiety-induced sleep disturbance among in-school adolescents in Ghana. (2) *Methods:* Analysis was performed using the 2012 Global School-based Health Survey (GSHS). A sample of 1342 in-school adolescents was included in the analysis. The outcome variable was anxiety-induced sleep disturbance reported during the past 12 months. Frequencies, percentages, chi-square, and multivariable logistic regression analyses were conducted. Results from the multivariable logistic regression analysis were presented as crude and adjusted odds ratios at 95% confidence intervals (CIs) and with a statistical significance declared at *p* < 0.05. (3) *Results:* Adolescents who went hungry were more likely to report anxiety-induced sleep disturbance compared to their counterparts who did not report hunger (aOR = 1.68, CI = 1.10, 2.57). The odds of anxiety-induced sleep disturbance were higher among adolescents who felt lonely compared to those that never felt lonely (aOR = 2.82, CI = 1.98, 4.01). Adolescents who had sustained injury were more likely to have anxiety-induced sleep disturbance (aOR = 1.49, CI = 1.03, 2.14) compared to those who had no injury. Compared to adolescents who never had suicidal ideations, those who reported experiencing suicidal ideations had higher odds of anxiety-induced sleep disturbance (aOR = 1.68, CI = 1.05, 2.71). (4) *Conclusions:* Anxiety-induced sleep disturbance among in-school adolescents were significantly influenced by the psychosocial determinants such as hunger, loneliness, injury, and suicidal ideation in this study. The findings can help design appropriate interventions through effective strategies (e.g., early school-based screening, cognitive-behavioral therapy, face-face counseling services) to reduce psychosocial problems among in-school adolescents in Ghana.

## 1. Introduction

Psychological problems of adolescents have become a global and safety concern [1]. Costello et al. [2] asserted that symptoms of anxiety are among the health problems affecting adolescents. According to the World Health Organization [3], “mental health conditions account for 16% of the global burden of disease and injury in people aged 10–19 years”. About 10–20% of adolescents worldwide experience mental health conditions [4]. However, these health conditions are often undetected and untreated [4,5,6]. As is argued, most in-school adolescents experience mental health conditions through psychological distress from sexual and social relations decisions and assignment/examination worries [7,8,9]. 

Globally, “anxiety is the ninth leading cause of illness and disability for adolescents aged 15–19 and sixth for those aged 10–14” [3]. In sub-Saharan Africa (SSA), depression and anxiety are the most common mental disorders [10,11]. A wide range of symptoms of anxiety-induced sleep disturbance in adolescents include sleeplessness, restlessness, intermittent nighttime awakenings, and fatigue as a result of different forms of anxiety disorders: generalized anxiety disorder, separation anxiety disorder, panic disorder, social phobia, specific phobia, obsessive-compulsive disorder, and posttraumatic or acute stress disorder [12,13]. Anxiety-induced sleep disturbance refers to poor sleep quality, clinical insomnia, and/or alterations or deficits in sleep as a result of loneliness (either social or emotional), worry, sleep disturbance, and excessive fear [13,14,15,16]. Parekh [13] argues that anxiety can signal us of any danger and then helps us to deal with it. However, some adolescents who experience symptoms of anxiety resort to unacceptable behavior (such as suicide, fights, and stealing), tobacco use and consumption of illicit drugs [14,17]. 

Empirical evidence has shown that adolescents’ mental health conditions include depression, anxiety, emotional disorders, post-traumatic stress, and psychosocial distress [1,7,18,19,20]. However, research on anxiety-induced sleep disturbance among in-school adolescents has received less attention, particularly in low- and middle-income countries. Thus, there is little understanding of the determinants of anxiety-induced sleep disturbance in these countries, particularly in sub-Saharan African countries. Few studies on anxiety-induced sleep disturbance among in-school adolescents have shown the prevalence and its correlates [1,16,21,22,23]. For instance, Khan and Khan [1] reported 4.7% of anxiety among adolescents in Bangladesh. Khan and associates further observed that anxiety significantly increases the likelihood of adolescents’ suicidal ideation, plans, and attempts as well as alcohol and drug abuse. A study conducted by Abbo et al., [21] in Uganda found 26.6% prevalence of anxiety among children aged 3–19 years. A similar study [22] has found a varied prevalence rate of anxiety among adolescents aged 13–18 years. 

Up until now, several factors (e.g., socio-demographic- age, school, or grade level; environmental stressors or psychosocial factors- alcohol/drug use, examination workload, loneliness, low self-esteem, victimization) have been found to be associated with anxiety-induced sleep disturbance among in-school adolescents [1,14,16,23,24,25,26]. However, these factors may vary in different populations. For example, in a study among in-school adolescents in Ghana, loneliness was reported among 18.4% of school-going adolescents in the 2012 Global School-based Health Survey [16]. Although the study found that anxiety was strongly associated with loneliness, it was used as an independent variable to check its prediction on loneliness. However, loneliness as a determinant of anxiety-induced sleep disturbance among in-school adolescents was not established. Thus, the predictors of anxiety-induced sleep disturbance of in-school adolescents in Ghana remain uncharted. This study, therefore, seeks to utilize data from the 2012 Global School-based Health Survey (GSHS) of Ghana to determine the factors that predict anxiety-induced sleep disturbance among in-school adolescents to fill this research gap. Findings of the study could provide useful information for researchers and policy direction related to the diagnosis and management of anxiety symptoms among adolescents in Ghana.

## 2. Materials and Methods

### 2.1. Study Design

This cross-sectional study used a secondary data from the 2012 GSHS. We relied on the “Strengthening the Reporting of Observational Studies in Epidemiology” (STROBE) statement in writing the manuscript. The GSHS is a nationwide study conducted among in-school adolescents to determine the risk and protective factors of health and behavioral problems [27]. The GSHS study was carried out in Ghana with support and assistance from the World Health Organization (WHO), the Centre for Disease Control and Prevention (CDC), and the Ministry of Education (MoE). A close-ended questionnaire was used to obtain the primary data. A two-staged probability sampling technique was used to recruit students from 25 senior high schools (SHS) across the then 10 regions in Ghana. First, study schools were proportionally selected based on the school’s population (enrolment size) using a systematic random sampling method. The second stage involved the random selection of study classes from each selected school. These sampling techniques used provide the avenue to generalize the results to in-school adolescents in a broader context. Moreover, the design and techniques employed offer the opportunity to subject the data to rigorous statistical analysis. A total of 1984 students from SHSs participated in the study. The overall, school and students’ response rates were 71%, 96%, and 74%, respectively. However, 1342 in-school adolescents with complete cases on all the variables of interest were included in the study. The dataset is available freely at https://www.who.int/ncds/surveillance/gshs/ghanadataset/en/. 

### 2.2. Study Variables

#### 2.2.1. Outcome Variable

The main outcome variable in the study was anxiety-induced sleep disturbance. This variable was derived from the question, “During the past 12 months, how often have you been so worried about something that you could not sleep at night?” The responses were 1 = never, 2 = rarely, 3 = sometimes, 4 = most of the times, and 5 = always. The responses were dichotomized into “no anxiety (0)” and “anxiety (1)”. If the student responded never/rarely/sometimes, the study respondent was then categorized as “not felt anxious” while those who responded as “most at times” or “always” were grouped as “felt anxious” [1]. 

#### 2.2.2. Explanatory Variables

A total of twenty-two explanatory variables were used in the present study. The variables were selected from a review of the literature [1] and their availability in the dataset. The variables included age, sex, grade, hunger, physical fighting, physical attack, injury, truancy, alcohol use, cigarette smoking, marijuana use, tobacco use, peer support, close friends, suicidal ideation, suicidal attempt, loneliness, bullied, parental or guardian supervision, parental or guardian bonding, parental or guardian connectedness, and parental or guardian respect for privacy. All the variables were recoded into a binary form, except grade. The detailed description of the variables and the recoded responses are in the supplementary file attached (Table S1).

### 2.3. Statistical Analyses

Data for this study was extracted, cleaned, recoded, and analyzed using Stata software version 16.0 (Stata Corporation, College Station, TX, USA). Variables with complete cases were included in the final analysis. Both descriptive and inferential analyses were performed. Descriptively, frequencies, and percentages were used to present the results. The inferential analysis was subdivided into two stages. The first stage consisted of a Chi-square test, conducted to examine the relationship between anxiety-induced sleep disturbance and explanatory variables. The statistically significant variables (i.e., showed a *p* < 0.05) were subsequently included in a multivariable analysis. At the second stage, multivariable binary logistic regression analysis was also carried out. The binary logistic regression model was used because the main outcome variable in the data was recoded to dichotomous which met the basic assumption for the use of binary logistic regression. Two regression models were used during the binary logistic regression analysis. The first model (Model I) was used to determine the strength of the association between each of the explanatory variables and anxiety-induced sleep disturbance. The second model (Model II) was built to examine the strength of the association between all the significant explanatory variables from the Chi-square analysis and anxiety-induced sleep disturbance. The results in the binary logistic regression analyses for Model I and Model II were presented using the crude odds ratios (cOR) and adjusted odds ratios (aOR) with their corresponding 95% confidence intervals (CIs). Statistical significance was set at *p* < 0.05. During the analysis, we adjusted for the complex design employed in the data collection. Multicollinearity was checked using the Variance Inflation Factor (VIF). A mean VIF of 1.24 shows no evidence of multicollinearity in the data.

### 2.4. Ethical Consideration

Ethical requirements for the use of both minors and adults were adhered to during the survey. First, institutional permission for the conduct of the study was sought from the MoE and Heads of selected schools. All the ethical guidelines and procedures regarding the conduct of research among students were strictly adhered to during the study. Prior to the data collection, written permission was sought from the Ghana Education Service (GES), heads, and teachers of the selected schools. Written informed consent was obtained from students aged 18 years and above. Child agreement was sought from students below 18 years. Additionally, a written parental or guardian consent was obtained from those whose wards were minors before their inclusion in the study. 

## 3. Results

### 3.1. Socio-Demographic Characteristics of the Respondents

Of the 1342 in-school adolescents who were included in the study, 97.2% were aged 15 years or older. More than half (55.2%) were males and the rest being females (44.8%). The majority (32.0%) of the adolescents were in SHS3, followed by SHS1 (29.5%), and lowest in SHS2 (18.8%). Only a few (12.1%) went hungry in the past 30 days before the study.

### 3.2. Bivariate Analysis of the Proportion of Anxiety-Induced Sleep Disturbance among Adolescents in Ghana

Table 1 shows the prevalence of anxiety-induced sleep disturbance and its distribution among the in-school adolescents in Ghana. The results showed that the prevalence of anxiety-induced sleep disturbance among the adolescents was 13.9%. In the Chi-square analysis, hunger (χ^2^ = 15.7, *p* < 0.001), loneliness (χ^2^ = 53.1, *p* < 0.001), suicidal ideation (χ^2^ = 19.4, *p* < 0.001), suicidal attempt (χ^2^ = 10.0, *p* < 0.001), bullied (χ^2^ = 9.5, *p* = 0.002), injured (χ^2^ = 17.2, *p* < 0.001), engaged in fight (χ^2^ = 4.3, *p* = 0.038), attacked (χ^2^ = 11.7, *p* = 0.001), and parental or guardian respect for privacy (χ^2^ = 7.5, *p* = 0.006) were significantly associated with anxiety-induced sleep disturbance among the in-school adolescents. 

### 3.3. Multivariable Regression Analysis on Predictors of Anxiety-Induced Sleep Disturbance among In-School Adolescents in Ghana

Table 2 presents the multivariable logistic regression results of the association between anxiety-induced sleep disturbance and the explanatory variables. The results from the crude odds ratio (Model I) showed that all the explanatory variables included from the bivariate analysis showed significant associations with anxiety-induced sleep disturbance. However, in the adjusted model (Model II), only ever went hungry, loneliness, injury, and suicidal ideation showed statistically significant associations with anxiety-induced sleep disturbance. Adolescents who went hungry were more likely to report anxiety-induced sleep disturbance compared to their counterparts who did not report hunger (aOR = 1.68, CI = 1.10, 2.57). The odds of anxiety-induced sleep disturbance were high among adolescents who felt lonely compared to those who never felt lonely (aOR = 2.82, CI = 1.98, 4.01). Adolescents who had sustained injury were more likely to have anxiety-induced sleep disturbance (aOR = 1.49, CI = 1.03, 2.14) compared to those who had no injury. Compared to adolescents who never had suicidal ideations, those who reported experiencing suicidal ideations had higher odds of anxiety-induced sleep disturbance (AOR = 1.68, CI = 1.05, 2.71). 

AOR-Adjusted Odds Ratio; COR-Crude Odds Ratio; CI-Confidence interval; Ref- Reference.

## 4. Discussion

This study investigated the factors associated withanxiety-induced sleep disturbance among in-school adolescents in Ghana using the 2012 GSHS. The results revealed that the prevalence of anxiety-induced sleep disturbance among in-school adolescents was 13.9%. Hunger, loneliness, suicidal ideation and injury are associated with an anxiety-induced sleep disturbance among in-school adolescents in Ghana. The prevalence of anxiety-induced sleep disturbance in Ghana is similar to what was reported in India [28], Uganda [21], Nigeria [22], and China [29]. Other countries such as Mexico and Bangladesh also reported lower prevalence rates [1,30]. Since most of the respondents were in their final year, it is more likely that they were overburdened with high expectations in the final examination from their parents, siblings, and friends. The prevalence rate of anxiety-induced sleep disturbance among the in-school adolescents in Ghana is a public health concern for school directors and the Ministry of Education. Hence, there is a pressing need to develop timely psychological interventions such as field trips [16,31], and effective counseling services in SHS in Ghana to drastically reduce anxiety among adolescents, especially during their exam periods. 

Corroborating a previous study [32], adolescents who went hungry were more likely to report anxiety-induced sleep disturbance than those who did not. Since most of the adolescents’ abuse and misuse drugs, and their incessant quest for food, would be experienced as a result of the drugs’ effect [24]. Additionally, there is a possibility that the adolescents might have used all their money on illicit drugs; hence, they would have nothing to spend on. Very crucially, the implementation of programs that would help intensify the education on the negative impacts of illicit drugs use in SHS in Ghana is laudable.

Lonely adolescents were more likely to report anxiety-induced sleep disturbance than those who did not, a finding that is consistent with several previous studies [23,24,31,33,34]. Adolescents who face parental occupational conflicts and come from low-income households with low purchasing power for family needs may experience intermittent loneliness which might trigger social anxiety. Similarly, loneliness could cause anxiety-induced sleep disturbance among adolescents because of inadequate support from friends, loved ones and other influential people who play significant roles in helping them to manage any stressful occurrences or situations they may be facing [1,24]. More importantly, adolescents who experience loneliness are often rejected by their peers. As a result, they find it difficult to share their problems with other people. Such adolescents are quite vulnerable to several anxiety-related health problems [35].

Similar to a previous study [36], adolescents who sustained injury were more likely to report anxiety-induced sleep disturbance than those who did not. An unexpected injury may evoke certain restrictions towards life emergencies and other activities of daily living. Such restrictions on one’s daily activities could create irrational thoughts and cause anxiety-induced sleep disturbance [7,37]. Also, any unintended injury to regular in-school adolescents that may require treatment for several days, weeks, or months in extreme cases might distort their academic pursuits [17,21]. In relation to this, any distortion of academic work may scare them, especially as their final examination gets closer. Such adolescents require appropriate self-support interventions that aim at increasing injured persons’ ability to independently handle their setbacks (e.g., sustaining an injury).

Other findings revealed that adolescents who experienced suicidal ideations were more likely to report anxiety-induced sleep disturbance compared to those who did not [17,35,37]. Most suicidal ideations manifest through persons with history of minor or major psychological, intrapersonal, or individual-level factors and/or social problems (e.g., psychiatric complications, physical illness, and social disconnectedness with loved ones- parents, siblings) [23,37,38,39]. Such persons usually experience various degree of anxiety disorders such as generalized anxiety disorder, separation anxiety disorder, panic disorder, social phobia, specific phobia, obsessive-compulsive disorder, and posttraumatic or acute stress disorder [12,13]. Therefore, giving enough privacy to adolescents could trigger suicidal ideations or other self-damaging acts [17,35]. This finding suggests that enhanced services and education on knowledge of risk and protective strategies for curbing suicidal ideation is needed in the various SHSs in Ghana.

### 4.1. Strengths and Limitations

Unlike any other research, this study has some strengths and limitations. The study relies on a unique data set that links data from standardized data collection through the global health survey. The use of questionnaires for the secondary data permitted the assessment of multiple determinants associated with anxiety-induced sleep disturbance. The relatively large sample size, selected using systematic random procedure with a high response rate warrants generalizability of findings to other homogenous populations, Despite these strengths, there are some limitations that need to be mentioned. First, the assessment of anxiety-induced sleep disturbance was based on self-reports, hence, may be subject to recall and social desirability bias though standardized procedures (e.g., clear and simple instructions) were used to minimize any potential for such biases. Second, the assessment of anxiety-induced sleep disturbance relied on a simple question pertaining to “worry” that may be influenced by several factors such as personality. Due to the cross-sectional design nature of the survey data, the predictors noted in this study are only from a logical perspective devoid of any causality. 

### 4.2. Practical Implications

This study provides useful information on the prevalence and predictors of anxiety-induced sleep disturbance among in-school adolescents in Ghana. Recognizing these determinants could help early identification and management of anxiety-induced sleep disturbance in these adolescents. Evidence suggests that anxiety-induced sleep disturbance warrant early detection and management because of the adverse psychological consequences (e.g., impaired cognitive/physical functioning and intra-personal and educational difficulties) among adolescents [40]. To reduce a potential public health burden of the country, the findings necessitate mental health intervention programs (e.g., early school-based screening, cognitive-behavioral therapy, and face-face counseling services) across SHSs in the country. Mental health policy on early detection and reduction of anxiety in SHS would be worthwhile. Future research could target the effectiveness of interventions implemented to help Ghanaian SHS students having anxiety disorders using longitudinal designs. 

## 5. Conclusions

Findings of this study indicate that the prevalence of anxiety-induced sleep disturbance among in-school adolescents in Ghana remains high, with hunger, loneliness, injury, and suicidal ideation as associated determinants. Considering the mental health challenges that anxiety-induced sleep disturbance may create for in-school adolescents, understanding the determinants could lead to early detection and management. These findings can help with the design of appropriate interventions through effective strategies (e.g., early school-based screening, cognitive-behavioral therapy, and face-face counseling services) to reduce psychosocial problems among in-school adolescents in Ghana. 

## Figures and Tables

**Table 1:** Bivariate Analysis of the Proportion of Anxiety-induced sleep disturbance among In-school Adolescents in Ghana.

Variable	N = 1342		Anxiety-Induced Sleep Disturbance		Chi-Square χ^2^ (*p*-Value)
	Frequency	Percent	No	Yes	
**Anxiety-induced sleep disturbance**			86.1	13.9	
**Age**					0.02 (0.899)
12–14 years	38	2.8	86.8	13.2	
15–18 years	1304	97.2	86.1	13.9	
**Sex**					0.8 (0.365)
Female	601	44.8	85.2	14.8	
Male	741	55.2	86.9	13.1	
**Grade**					0.7 (0.865)
SHS 1	396	29.5	86.1	13.9	
SHS 2	253	18.8	85.0	15.0	
SHS 3	429	32.0	87.2	12.8	
SHS 4	264	19.7	85.6	14.4	
**Ever went hungry**					15.7 (<0.001)
No	1179	87.9	87.5	12.5	
Yes	163	12.1	76.1	23.9	
**Loneliness**					53.1 (<0.001)
No	1100	82.0	89.4	10.6	
Yes	242	18.0	71.5	28.5	
**Truant**					0.5 (0.459)
No	933	69.5	86.6	13.4	
Yes	409	30.5	85.1	14.9	
**Suicidal ideation**					19.4 (<0.001)
No	1134	84.5	87.9	12.1	
Yes	208	15.5	76.4	23.6	
**Suicidal attempt**					10.0 (0.002)
No	1081	80.6	87.6	12.4	
Yes	261	19.4	80.1	19.9	
**Alcohol use**					1.2 (0.264)
No	1187	88.5	86.5	13.5	
Yes	155	11.5	83.2	16.8	
**Tobacco use**					0.7 (0.397)
No	1279	95.3	86.3	13.7	
Yes	63	4.7	82.5	17.5	
**Smoking**					0.1 (0.780)
No	1303	97.1	86.2	13.8	
Yes	39	2.9	84.6	15.4	
**Marijuana use**					0.1 (0.822)
No	1310	97.6	86.1	13.9	
Yes	32	2.4	87.5	12.5	
**Bullied**					9.5 (0.002)
No	788	58.7	88.6	11.4	
Yes	554	41.3	82.7	17.3	
**Injury**					17.2 (<0.001)
No	651	48.5	90.2	9.8	
Yes	691	51.5	82.3	17.7	
**Engaged in fight**					4.3 (0.038)
No	972	72.4	87.3	12.7	
Yes	370	27.6	83.0	17.0	
**Attacked**					11.7 (0.001)
No	857	63.9	88.6	11.4	
Yes	485	36.1	81.9	18.1	
**Number of close friends**					0.1 (0.782)
No	179	13.3	85.5	14.5	
Yes	1163	86.7	86.2	13.8	
**Peer support**					0.5 (0.465)
No	870	64.8	85.6	14.4	
Yes	472	35.2	87.1	12.9	
**Parental or guardian supervision**					0.2 (0.683)
No	776	57.8	86.5	13.5	
Yes	566	42.2	85.7	14.3	
**Parental or guardian connectedness**					0.6 (0.457)
No	731	54.5	85.5	14.5	
Yes	611	45.5	86.9	13.1	
**Parental or guardian bonding**					0.02 (0.892)
No	802	59.8	86.0	14.0	
Yes	540	40.2	86.3	13.7	
**Parental or guardian respect for privacy**					7.5 (0.006)
No	1130	84.2	87.3	12.7	
Yes	212	15.8	80.2	13.7	

Source: GSHS, 2012.

**Table 2:** Multivariable Analysis on Predictors of Anxiety-induced sleep disturbance among In-school Adolescents in Ghana.

Variable	Anxiety-Induced Sleep Disturbance	
	Model I COR (95% CI) *p*-Value	Model II AOR (95% CI) *p*-Value
**Ever went hungry**		
No	Ref	Ref
Yes	2.21 (1.48, 3.29) <0.001	1.68 (1.10, 2.57) 0.016
**Loneliness**		
No	Ref	Ref
Yes	3.35 (2.39, 4.70) <0.001	2.82 (1.98, 4.01) <0.001
**Bullied**		
No	Ref	Ref
Yes	1.63 (1.19, 2.22) 0.002	1.09 (0.77, 1.55) 0.621
**Injury**		
No	Ref	Ref
Yes	1.97 (1.42, 2.72) <0.001	1.49 (1.03, 2.14) 0.032
**Engaged in fight**		
No	Ref	Ref
Yes	1.42 (1.02, 1.97) 0.039	0.98 (0.67, 1.43) 0.911
**Attacked**		
No	Ref	Ref
Yes	1.72 (1.26, 2.35) 0.001	1.38 (0.97, 1.96) 0.077
**Suicidal ideation**		
No	Ref	Ref
Yes	2.24 (1.55, 3.24) <0.001	1.68 (1.05, 2.71) 0.031
**Suicidal attempt**		
No	Ref	Ref
Yes	1.76 (1.23, 2.50) 0.002	0.93 (0.77, 1.47) 0.744
**Parental or guardian respect for privacy**		
No	Ref	Ref
Yes	1.69 (1.16, 2.47) 0.007	1.41 (0.94, 2.11) 0.097
N		1342
Pseudo R^2^		0.0734

Source: GSHS, 2012.

## Data Availability

The dataset is available for free at: https://www.who.int/ncds/surveillance/gshs/ghanadataset/en/.

## Supplementary Materials

The following are available online at https://www.mdpi.com/2076-328X/11/2/20/s1, Table S1: Study variables.
