# Supplementary material for: Predictors of Anxiety-Induced Sleep Disturbance among in-School Adolescents in Ghana: Evidence from the 2012 Global School-Based Health Survey
Source: Behav Sci (Basel). 2021 Feb 1;11(2):20. doi: 10.3390/bs11020020 (PMC7912318; doi:10.3390/bs11020020)
Supplement: Supplementary file 1 [file behavsci-11-00020-s001.pdf]

Table S1. Study variables

Variables	Question	Response options and recoding
**Outcome variable**		
Anxiety-induced sleep disturbance	During the past 12 months, how often have you been so worried about something that you could not sleep at night?	1 = never to 5 = always (coded 1 - 3 = 0, 4 – 5 = 1)
**Explanatory variables**		
Socio-demographic characteristics		
Age	How old are you?	1=12, 2=13, 3=14, 4=15, 5=16, 6=17, 7=18 years (coded as 0=1-14, 15-19)
Sex	What is your sex?	1=male, 2=female (coded 2=0, 1=male)
Grade	In what grade are you?	1=SHS1, 2=SHS2, 3=SHS3, 4=SHS4
Hunger (proxy of socioeconomic status)	Went hungry past 30 days	1=never, 2=Rarely, 3=sometimes, 4=most of the times, 5=always (coded 1-3=0, 4-5=1)
Psychosocial environmental factors		
Tobacco use	During the past 30 days, on how many days did you use any other form of tobacco, such as chewing tobacco leaves?	1 = 0 days; to 7 = All 30 days (coded as 1 = 0; and 2–7 = 1)
Alcohol use	During the past 30 days, on how many days did you have at least one drink containing alcohol?	1 = 0 days; to 7 = All 30 days (coded as 1 = 0; and 2–7 = 1)
Smoking	During the past 30 days, how many days did you smoke cigarette?	1 = 0 days; to 7 = All 30 days (coded as 1 = 0; and 2–7 = 1)
Marijuana use	During the past 30 days, how many times have you used marijuana (also called wee, Jah, Indian hemp, ahabammono, and ganja)	1=0 times; to 5=20 or more times (coded as 1=0; and 2-5=1)
Loneliness	During the past 12 months, how often have you felt lonely?	1=never, 2=rarely, 3= sometimes, 4 = most of the time to 5 = always (coded as 1-3 = 0; and 4- 5 = 1)
Injury	During the past 12 months, how many times were you seriously injured?	1 = 0 times; to 8 = 12 or more time (coded as 1 = 0; and 2–8 = 1)
Truancy	During the past 30 days, on how many days did you miss classes or school without permission?	1=0 days,2= 1or 2 days, 3=3 to 5 days, 4=6 to 9 days, 5= 10 or more (coded as 1=0; and 2-5=1)
Suicidal ideation	During the past 12 months, did you ever seriously consider attempting suicide?”	1 = yes, 2 = no (coded 2 = 0; and 1 = 1)
Suicidal attempt	During the past 12 months, how many times did you actually attempt suicide?	1=0 times; to 5= 6 or more times (coded as 1=0; and 2-5=1)
Bullied	During the past 30 days, how were you bullied most often?	1 = 0 times; to 8 = 12 or more times (coded as 1 = 0; and 2–7 = 1)
Fight	During the past 12 months, how many times were you in a physical fight?	1 = 0 times; to 8 = 12 or more times (coded as 1 = 0; and 2–8 = 1)

Injury	During the past 12 months, how many times were you seriously injured?	1 = 0 times; to 8 = 12 or more time (coded as 1 = 0; and 2–8 = 1)
Attacked	During the past 12 months, how many times were you physically attacked?	1=0 days,2= 1or 2 days, 3=3 to 5 days, 4=6 to 9 days, 5= 10 or more (coded as 1=0; and 2-8=1)
Close friends	How many close friends do you have?	1=0 to 4=3 or more (coded as 1=0; and 2-4=1)
Helpful (Peer support)	During the past 30 days, how often were most of the students in your school kind and helpful?	1=never, 2=Rarely, 3=sometimes, 4=most of the times, 5=always (coded as 1-3 = 0; and 4–5 = 1)
Parents check homework (parental supervision)	During the past 30 days, how often did your parents or guardians check to see if your homework was done?	1=never, 2=Rarely, 3=sometimes, 4=most of the times, 5=always (coded as 1-3 = 0; and 4–5 = 1)
Understand problems (Parental Connectedness	During the past 30 days, how often did your parents or guardians understand your problems and worries?	1=never, 2=Rarely, 3=sometimes, 4=most of the times, 5=always (coded as 1-3 = 0; and 4–5 = 1)
Know what adolescent do free time (Parental or guardian Bonding)	During the past 30 days, how often did your parents or guardians really know what you were doing with your free time?	1=never, 2=Rarely, 3=sometimes, 4=most of the times, 5=always (coded as 1-3= 0; and 4–5 = 1)
Parental or guardian respect for Privacy	During the past 30 days, how often did your parents or guardians go through your things without your approval?	1 = never; to 5 = always (coded as 1-3= 0; and 4-5 = 1)
